# Targeting the Highly Deleterious G161C and Y260C SNP Variants of the AGXT Protein Involved in Glyoxylate Metabolism Using Tauroursodeoxycholic Acid: A Computational Study

**DOI:** 10.3390/ijms27104590

**Published:** 2026-05-20

**Authors:** Shruthika Giridharan, Vasundra Vasudevan, Sidharth Kumar Nanda Kumar, Madhana Priya Nanda Kumar, Magesh Ramasamy

**Affiliations:** 1 Department of Biotechnology, Faculty of Biomedical Sciences & Technology, Sri Ramachandra Institute of Higher Education and Research (DU), Chennai 600116, India; shruthikagiridharan@gmail.com (S.G.); vachuvasudev@gmail.com (V.V.); sidharthnandakumar01@gmail.com (S.K.N.K.); 2 Center for Transdisciplinary Research, Department of Pharmacology, Saveetha Dental College and Hospitals, Saveetha Institute of Medical and Technical Sciences, Chennai 600077, India

**Keywords:** *AGXT*, PH1, neglected disease, healthcare, SNP variants, molecular docking, molecular dynamics, FEL, DCCM, TUDCA

## Abstract

Hyperoxaluria Type 1 (PH1) is a rare autosomal recessive metabolic disorder caused by mutations in the *AGXT* gene, leading to impaired glyoxylate metabolism and excessive oxalate accumulation, resulting in nephrolithiasis, nephrocalcinosis, and end-stage renal disease. As a rare and often neglected disease, PH1 poses a significant challenge to modern healthcare systems due to its progressive nature and limited therapeutic options. In this study, an integrated in silico approach was employed to identify pathogenic single-nucleotide polymorphisms (SNPs) and evaluate potential therapeutic candidates. Computational analyses using ConSurf, Align-GVGD, INPS-MD, CUPSAT, and iStable identified G161C and Y260C as highly deleterious variants affecting protein stability. Virtual screening, followed by ADME and toxicity assessments, identified Tauroursodeoxycholic acid (TUDCA) as a promising candidate with favorable pharmacokinetic and safety profiles. Molecular docking revealed that TUDCA exhibited higher binding affinity than the reference drug pyridoxine across native and SNP variants of AGXT proteins. Molecular dynamics simulations (300 ns) demonstrated enhanced structural stability of TUDCA-bound complexes, indicated by reduced RMSD and RMSF, improved compactness, and sustained hydrogen bonding. Furthermore, free energy landscape (FEL) and dynamic cross-correlation matrix (DCCM) analyses confirmed improved conformational stability and coordinated residue motions in SNP variant structures. Overall, these findings suggest that TUDCA may effectively stabilize structural alterations induced by pathogenic *AGXT* variants, highlighting its potential as a precision medicine-based therapeutic strategy for PH1.

## 1. Introduction

Primary hyperoxalurias (PH) are rare genetic disorders, of which 3 distinct types have been recognized to date, resulting in the formation of the most severe form of calcium oxalate (CaOx) kidney stone disease. All three forms of PH are autosomal recessive (AR) disorders resulting in the excessive formation of oxalate in the body [1]. PH type 1 (PH1) is the most common and most studied form. It is estimated that the prevalence of primary hyperoxaluria (PH) is less than 3 in 1,000,000, with PH1 making up about 80% of individuals diagnosed with PH [2].

**Figure 1:**
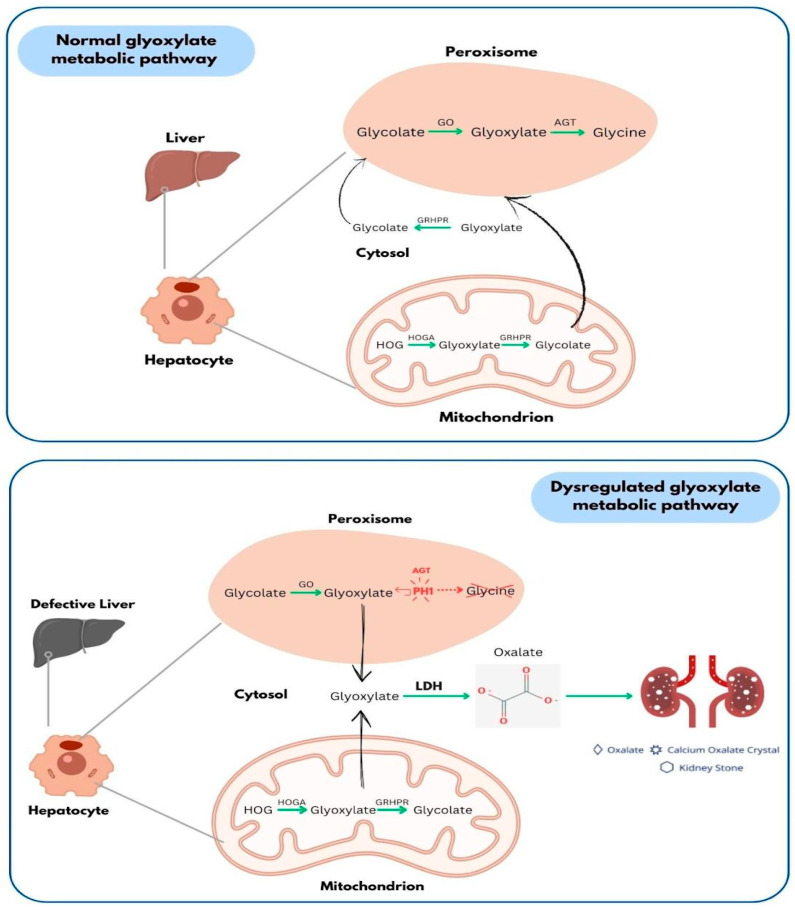
Schematic representation of glyoxylate metabolism and its disruption in Primary Hyperoxaluria Type 1 (PH1), highlighting key enzymes involved in glyoxylate detoxification and the accumulation of oxalate due to enzymatic defects. Green arrows indicate metabolic conversion pathways. Red boxes highlight key enzymes and affected components involved in the pathway. The dashed red box represents impaired enzymatic activity associated with mutations in the *AGXT* gene. The mitochondrial region illustrates alternative pathways involving enzymes such as HOGA1 and GRHPR.

PH1 is a genetic disorder inherited in an autosomal recessive pattern, characterized by the overproduction of oxalate leading to the abnormal accumulation of calcium oxalate, presenting as different forms of urolithiasis, nephrocalcinosis, and systemic oxalosis (SO) [3]. This condition is caused by mutations in the *AGXT* gene, which result in a deficiency of the enzyme alanine-glyoxylate aminotransferase (AGT), which is responsible for converting glyoxylate into glycine in the liver [4]. Consequently, glyoxylate builds up and is instead transformed primarily into oxalate, which is excreted via the kidneys (Figure 1).

The *AGXT* gene, located on chromosome 2q37.3, encodes for the enzyme alanine-glyoxylate aminotransferase (AGT), which is predominantly found in liver peroxisomes. AGT is essential for detoxifying glyoxylate by converting it into glycine. Mutations in the *AGXT* gene can disrupt the enzyme’s function, stability, and localization. Due to these genetic changes, there is insufficient or misdirected AGT, resulting in the accumulation of glyoxylate, which is then converted into oxalate [5]. In humans, oxalate is not further metabolized, and this excess oxalate interacts with calcium to create insoluble calcium oxalate (CaOx) crystals, potentially leading to kidney stones, nephrocalcinosis, and progressive renal injury, which may ultimately result in kidney failure [5]. The eventual decline in kidney function and the kidney’s reduced ability to eliminate oxalate from the bloodstream can lead to systemic oxalosis, characterized by the accumulation of oxalate in plasma and the formation of extrarenal CaOx crystals, causing damage to various organs, including bone, heart, skin, and eyes [6].

**Figure 2:**
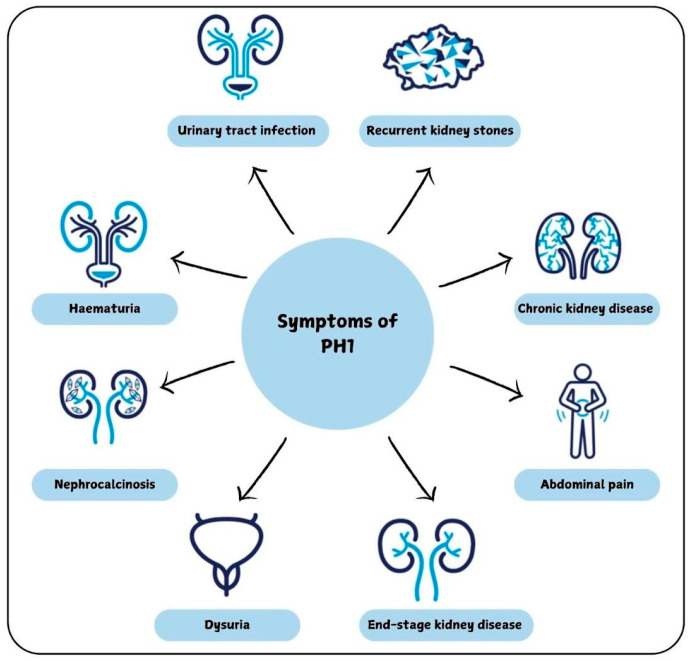
Clinical manifestations of Primary Hyperoxaluria Type 1 (PH1).

PH1 presents with a wide spectrum of clinical manifestations, ranging from early-onset kidney failure with systemic oxalosis in infants to recurrent nephrolithiasis and nephrocalcinosis in later stages. The disease is driven by excessive calcium oxalate accumulation, leading to progressive renal impairment and, in severe cases, systemic involvement affecting organs such as the eyes, heart, and bones (Figure 2). In some cases, PH1 is diagnosed only after recurrent kidney failure following transplantation. Notably, more than 70% of affected individuals progress to kidney failure, often requiring dialysis or transplantation [6].

Currently, the treatment options for PH1 are limited, primarily consisting of symptomatic therapies that can only postpone the onset of end-stage renal disease (ESRD). Hence, the only proven and effective treatment for PH1 is a dual liver–kidney transplant. The treatment approach for treating PH1 aims at maintaining kidney function and controlling CaOx buildup. There are established traditional methods in practice as well as new therapeutic strategies under investigation, among which targeted therapy rooted in the disease’s underlying mechanisms has emerged as a promising area of research for PH1 treatment [7].

Increased fluid intake, high doses of pyridoxine (which serves as a cofactor for AGT), and inhibitors of calcium oxalate crystallization, like citrate, might reduce the frequency of kidney stone formation and help in slowing down the progression of the disease. In severe situations, aggressive hemodialysis, occasionally combined with peritoneal dialysis, is necessary to eliminate excess oxalate. Liver transplantation addresses the underlying metabolic issue, normalizes oxalate levels, and can avert end-stage kidney disease if done in a timely manner. Lumasiran, a novel RNA interference (RNAi) therapy in development, decreases hepatic oxalate production by blocking glycolate oxidase, which raises glycolate levels for safe elimination, presenting a promising targeted approach for PH1 [8].

Computer-Aided Drug Discovery (CADD) is an in silico approach that integrates molecular modeling, virtual screening, and docking simulations to efficiently design and optimize potential drug candidates [9]. In this context, identifying highly deleterious *AGXT* variants that significantly disrupt protein stability is crucial for understanding disease severity and therapeutic targeting. Furthermore, exploring compounds capable of stabilizing mutation-induced structural alterations through a chemical chaperone-based approach may provide a promising strategy for mutation-specific intervention.

This study is therefore aimed at identifying critical SNP variants and evaluating potential stabilizing compounds using an integrated computational framework. SNP analysis was performed to identify SNP variants in the AGXT protein that may affect its function and interactions. Subsequently, molecular docking and molecular dynamics simulations were employed to assess the impact of these variants on protein stability and ligand binding, providing insights into precision medicine approaches and targeted therapeutic strategies.

## 2. Results

### 2.1. Dataset Retrieval for AGXT Protein

The AGXT protein structure (PDB ID: 5F9S; resolution 1.70 Å) was selected for analysis (Table 1). Energy minimization of the wild-type structure resulted in a final energy value of −18,907.004 kJ/mol, indicating a stable conformation suitable for further computational studies. A total of 261 SNP variants associated with the AGXT protein were compiled from multiple databases (Table 2).

Structural models of the selected variants, G161C and Y260C, were successfully generated and optimized. The minimized structures exhibited final energy values of −18,970.904 kJ/mol (G161C) and −18,835.734 kJ/mol (Y260C), confirming their structural stability. Binding site analysis identified key active site residues forming a prominent binding pocket, which was used for subsequent molecular docking studies (Table 3).

### 2.2. Evolutionary Analysis Results

**Figure 3:**
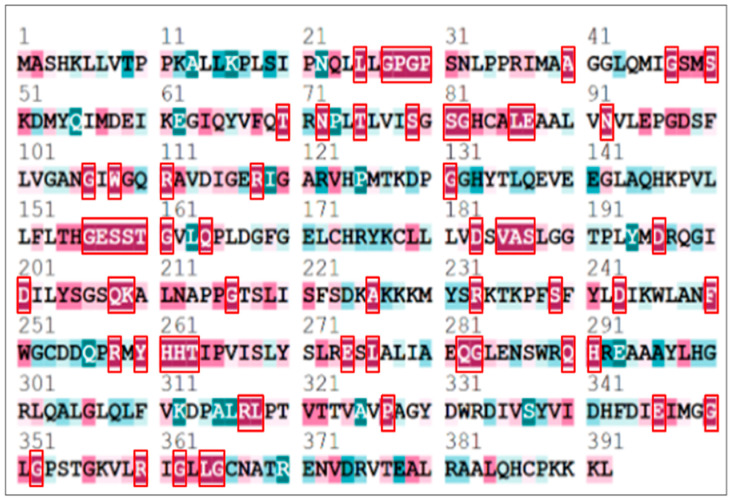
Analysis of evolutionary conservation of AGXT protein residues with the ConSurf program. Highly conserved residues (scoring 9) are highlighted in a red box, signifying their probable functional and structural significance.

The conserved residues of the AGXT protein were analyzed using the ConSurf tool. The protein’s FASTA sequence (392 amino acids) was employed along with homologous sequences retrieved from related species to assess evolutionary conservation. The ConSurf tool used this alignment to generate a conservation score ranging from 0 to 9, with 9 representing the most conserved residues. The analysis identified 77 highly conserved residues with a score of 9, which were then selected for further in silico predictions (Figure 3).

### 2.3. Biophysical Characterization

The biophysical characteristics of the SNP variants were analyzed using Align-GVGD. Among the 77 SNP variants found in conserved regions, 49 SNP variants were categorized as Class 65, suggesting a significant risk of functional disruption. Align-GVGD classifies variants according to their anticipated effects on protein functionality, with Class 65 denoting the most deleterious group. These SNP variants were taken for subsequent analyses (Table S1).

### 2.4. Stability Prediction Results

The structural stability of the SNP variants was evaluated using computational tools such as INPS-MD, CUPSAT, and iStable, focusing on the AGXT protein structure. The analysis revealed that some variants exhibited destabilizing effects on protein stability, while others maintained or enhanced stability. Among the 49 SNP variants analyzed biophysically, INPS-MD identified 11 SNP variants as destabilizing based on free energy estimations (Table S2). Of these, four SNP variants (S81W, W108C, G161C, and Y260C) were further identified as destabilizing by CUPSAT through structural analysis of atomic interactions and torsion angles (Table 4). Subsequent validation using iStable narrowed these findings to two highly deleterious SNP variants, G161C and Y260C, which were identified as the most deleterious SNP variants (Table 5).

### 2.5. Virtual Screening Results

Virtual screening was conducted using PyRx software 1.1. to identify potential drug candidates that could bind effectively to the AGXT protein. A total of 111 FDA-approved drug molecules, retrieved from the DrugBank database (https://go.drugbank.com/, accessed on 20 January 2026), were screened against the refined AGXT protein structure. Candidate selection was based on binding affinity and RMSD values, with compounds exhibiting RMSD of zero considered to have stable and reliable docking conformations. The top 50 compounds were shortlisted for further analysis (Table S3).

### 2.6. ADME Analysis Results

The drug-likeness of the selected compounds was evaluated using SwissADME on the basis of Lipinski’s Rule of Five. ADME evaluation was performed on the top 50 compounds from virtual screening. Of these, 32 compounds showed no violations of Lipinski’s rule, indicating them to be potential drug candidates (Table S4). These compounds were subsequently subjected to toxicity evaluation to exclude unsuitable candidates for further drug development.

### 2.7. Toxicity Prediction Results

Mcule and ProTox-3 tools were used to evaluate the toxicity of the 32 compounds that passed the ADME screening. MCULE toxicity prediction classified the compounds into 18 non-toxic and 15 toxic candidates, with non-toxic compounds prioritized for further analysis (Table S5). ProTox-3 toxicity prediction classified the compounds into different toxicity classes based on LD_50_ values, with 7 compounds in Class 5 and 2 compounds in Class 6, indicating low toxicity (Table 6). Among these, Tauroursodeoxycholic acid (PubChem ID: 9848818) was identified as the top candidate and selected for subsequent molecular docking based on its favorable toxicity profile.

### 2.8. Molecular Docking Results

Molecular docking analysis was conducted utilizing AutoDock 4 (The Scripps Research Institute, La Jolla, CA, USA) to assess the binding affinities of Tauroursodeoxycholic acid (the test compound) and Pyridoxine (the reference drug) with both the native (wild-type) and SNP Variants (G161C and Y260C) of the AGXT proteins (Protein ID: 5F9S). From the docking studies, it was clear that Tauroursodeoxycholic acid had a higher binding affinity than Pyridoxine for all protein variants. In particular, the wild-type protein exhibited a docking score of −6.43 kcal/mol with the TUDCA compound, indicating a stronger binding when compared to the reference compound’s score of −2.96 kcal/mol (Table 7). For the SNP variant G161C, Tauroursodeoxycholic acid demonstrated the highest binding affinity at −7.58 kcal/mol, further showing higher binding affinity compared to the reference compound. Similarly, the Y260C SNP variant, Tauroursodeoxycholic acid, exhibited enhanced binding (−5.24 kcal/mol) relative to Pyridoxine (−4.97 kcal/mol), underscoring its potential as a drug candidate.

**Figure 4:**
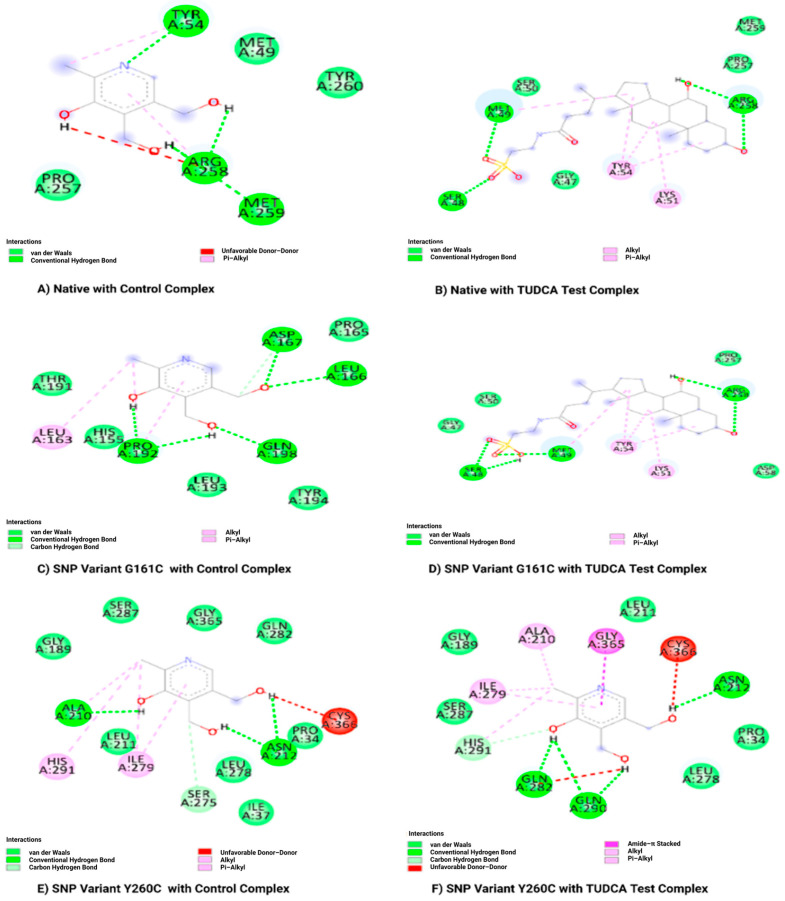
Docking interactions of native protein and SNP variants (G161C and Y260C) with reference compound and TUDCA test compound.

Molecular docking analysis demonstrated that the native protein forms stable interactions with both the reference drug and TUDCA test compound, especially engaging significant residues such as ARG258, MET259, and TYR54. The G161C SNP variant exhibited minor changes in binding with the reference drug; still, TUDCA sustained stable interactions, demonstrating considerable flexibility (Figure 4). The Y260C variant demonstrated significant structural alterations attributable to CYS266, influencing reference ligand binding, while TUDCA displayed robust and persistent interactions with residues such as GLN282, ASN212, and SER287 (Figure 4). TUDCA exhibited consistent binding to both native and variant proteins, indicating its potential to maintain efficacy despite structural differences caused by SNPs.

### 2.9. Molecular Dynamics Results

**Figure 5:**
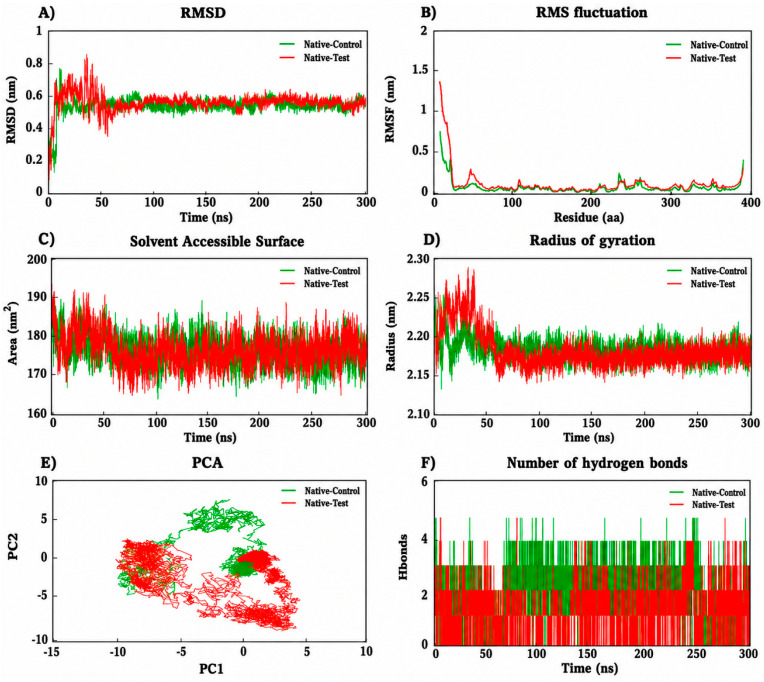
Molecular dynamics simulation analyses of the native protein in complex with reference drug (control) and test ligand over 300 ns: (**A**) root mean square deviation (RMSD), (**B**) root mean square fluctuation (RMSF), (**C**) solvent accessible surface area (SASA), (**D**) radius of gyration (Rg), (**E**) principal component analysis (PCA), and (**F**) number of hydrogen bonds, illustrating the structural stability, flexibility, compactness, conformational dynamics, and interaction stability of the complexes.

The 300 ns molecular dynamics simulation of the native *AGXT* complexes exhibited consistent structural stability throughout the simulation (Figure 5). RMSD analysis confirmed that both the reference drug (pyridoxine) and test (TUDCA) systems reached equilibrium during the initial 30–50 ns and maintained stability afterward, with average variances of approximately 0.5–0.6 nm (Figure 5A). The TUDCA-bound complex revealed comparable stability with slightly better trajectories, suggesting improved ligand accommodation and binding stability. RMSF analysis indicated minimal residue-level changes (<0.2 nm), with increased flexibility limited to terminal and loop regions, hence confirming the maintenance of structural integrity (Figure 5B). The radius of gyration (~2.18–2.22 nm) remained stable, showing sustained compactness (Figure 5C), while the solvent-accessible surface area (SASA) (~175–185 nm^2^) revealed no significant conformational expansion (Figure 5D). Principal component analysis (PCA) (Figure 5E) revealed a more compact and confined clustering of conformations in the TUDCA-bound complex compared to the reference drug, indicating reduced large-scale motions and enhanced conformational stability. PCA indicated that TUDCA induced considerable conformational sampling, demonstrating adaptive yet stable dynamics. Significantly, hydrogen bond studies revealed that the native TUDCA complex continuously maintained approximately 2 to 4 hydrogen bonds, equivalent to or slightly above the reference drug, indicating persistent intermolecular interactions (Figure 5F).

**Figure 6:**
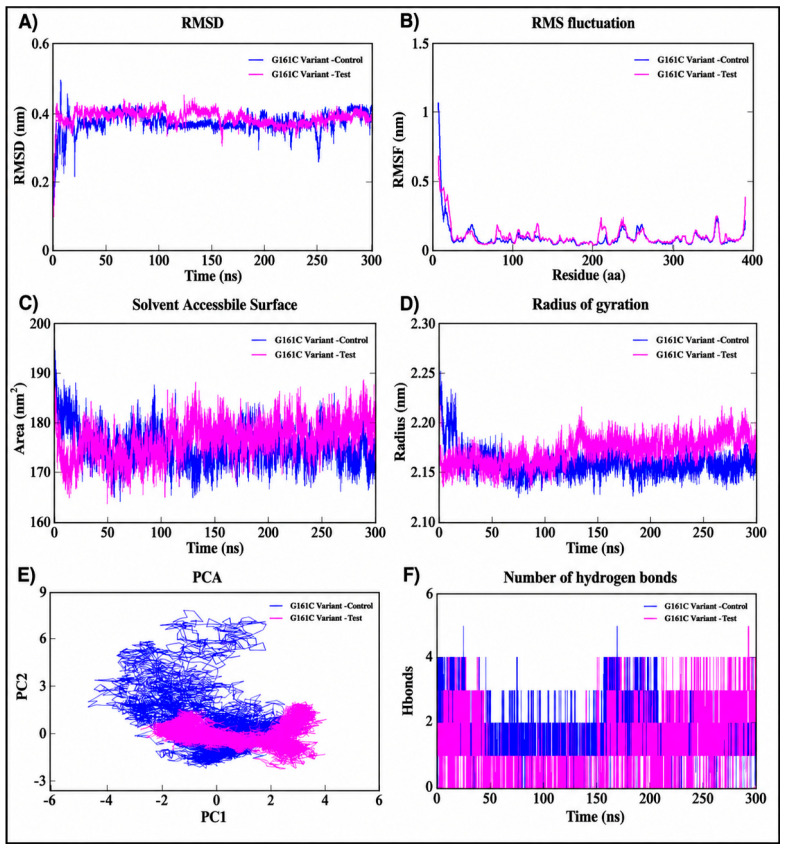
Molecular dynamics simulation analyses of the SNP variant G161C in complex with reference drug (control) and test ligand over 300 ns: (**A**) root mean square deviation (RMSD), (**B**) root mean square fluctuation (RMSF), (**C**) solvent accessible surface area (SASA), (**D**) radius of gyration (Rg), (**E**) principal component analysis (PCA), and (**F**) number of hydrogen bonds, illustrating the stability, flexibility, compactness, conformational dynamics, and interaction behavior of the SNP Variant complexes.

The 300 ns molecular dynamics simulation of the G161C SNP variant complex demonstrated significant stability in the presence of TUDCA (Figure 6). The RMSD stabilized after around 30–50 ns with reduced variations (approximately 0.35–0.45 nm), signifying enhanced structural stability relative to the native system. The TUDCA-bound complex exhibited diminished fluctuations and enhanced convergence (Figure 6A). RMSF analysis revealed negligible flexibility beyond terminal regions (Figure 6B). The radius of gyration (~2.15–2.20 nm) indicated increased compactness (Figure 6C), while the solvent-accessible surface area (SASA) (~170–180 nm^2^) showed less solvent exposure, underscoring structural restriction (Figure 6D). Principal component analysis (Figure 6E) revealed that the TUDCA-bound complex exhibited more compact conformational clustering compared to the pyridoxine-bound complex, indicating enhanced structural stability. PCA revealed more compact clustering for the TUDCA complex, signifying little conformational movement. Hydrogen bond research indicated that the TUDCA-bound variant sustained approximately 3–5 hydrogen bonds during the simulation, surpassing the reference drug system, which suggests enhanced and more stable ligand–protein interactions (Figure 6F).

**Figure 7:**
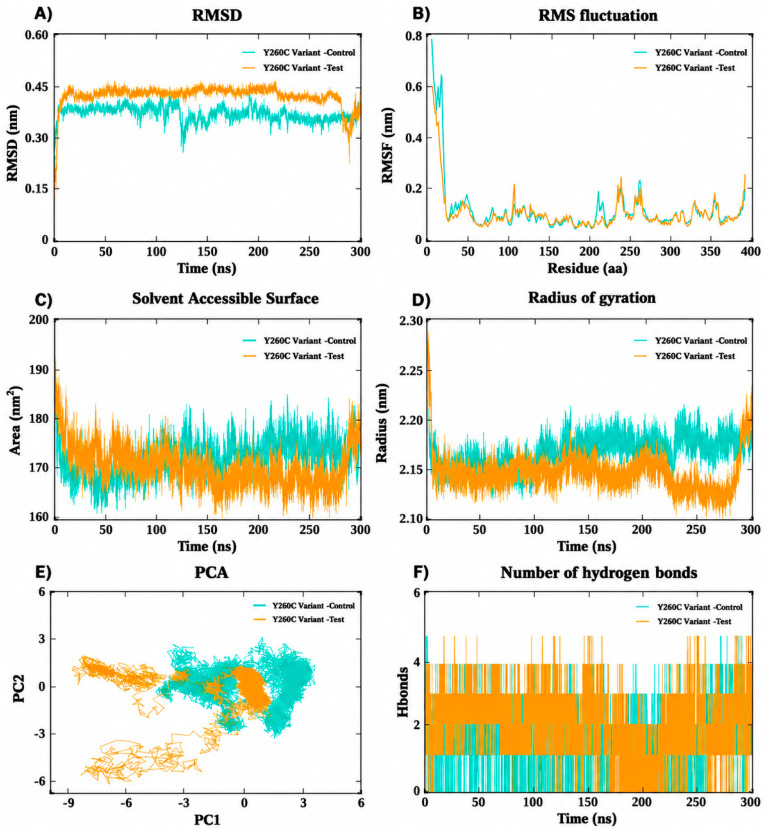
Molecular dynamics simulation analyses of the SNP variant Y260C in complex with reference and test ligands over 300 ns: (**A**) root mean square deviation (RMSD), (**B**) root mean square fluctuation (RMSF), (**C**) solvent accessible surface area (SASA), (**D**) radius of gyration (Rg), (**E**) principal component analysis (PCA), and (**F**) number of hydrogen bonds, illustrating the stability, flexibility, compactness, conformational dynamics, and interaction behavior of the SNP Variant complexes.

The 300 ns molecular dynamics simulation of the Y260C SNP variant demonstrated steady dynamics throughout the journey (Figure 7). RMSD values stabilized at about 50 ns (approximately 0.35–0.45 nm), with the TUDCA-bound complex exhibiting marginally enhanced stability relative to the reference drug (Figure 7A). The RMSF study showed minimal variation generally, with slight increases observed in the loop regions (Figure 7B). The radius of gyration (~2.15–2.22 nm) indicated stable compact folding (Figure 7D), and the solvent-accessible surface area (SASA) (~168–180 nm^2^) implied constant solvent exposure with a minor decrease in the TUDCA test complex (Figure 7C). PCA demonstrated more restricted clustering in the TUDCA-bound test complex (Figure 7E). Analysis of hydrogen bonds indicated that the TUDCA complex continuously sustained about 2 to 4 hydrogen bonds, hence ensuring robust interaction dynamics throughout the simulation (Figure 7F).

The 300 ns MD simulations indicate that the TUDCA test complex markedly improves the stability of both normal and variant AGXT proteins. Significant results include decreased RMSD, decreased residue fluctuations, enhanced compactness (Rg), reduced SASA, more cohesive PCA clustering, and augmented hydrogen bonding interactions relative to pyridoxine. TUDCA demonstrated around 2 to 4 hydrogen bonds in the native complexes, 3 to 5 hydrogen bonds in G161C variant complexes (indicating the maximum stability), and 2 to 4 hydrogen bonds in Y260C variant complexes, underscoring its robust and persistent binding affinity. The G161C variation had the most significant stabilizing impact, as TUDCA substantially mitigated mutation-induced instability. The findings robustly endorse TUDCA as an effective stabilizing ligand with mutation-specific treatment efficacy for Primary Hyperoxaluria Type 1.

### 2.10. FEL Analysis Results

**Figure 8:**
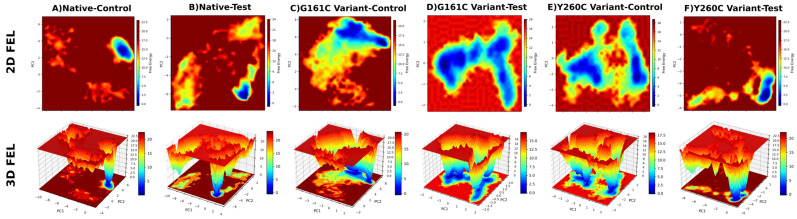
2D and 3D free energy landscape (FEL) plots of native and SNP variants: (**A**) Native-Control, (**B**) Native-Test, (**C**) G161C variant-Control, (**D**) G161C variant-Test, (**E**) Y260C variant-Control, and (**F**) Y260C variant-Test, showing conformational stability and energy distribution of the systems.

The free energy landscape (FEL) analysis, constructed by projecting molecular dynamics trajectories onto the first two principal components (PC1 and PC2), revealed distinct patterns of conformational stability among the native and SNP variant complexes (Figure 8). The FEL was generated by binning the conformational space and calculating free energy using the Boltzmann relation $G=-k_{B}TlnP$, where P represents the probability of each conformational state.

The native–pyridoxine (reference) complex displayed a distinct global minimum with a concentrated energy basin, indicating a stable conformational state, whereas the native–TUDCA complex exhibited a slightly broader distribution, suggesting moderate conformational flexibility. In the G161C variant, both complexes showed broader and more dispersed energy basins, reflecting increased structural fluctuations compared to the native system. However, the G161C–TUDCA complex demonstrated more confined minima than the pyridoxine-bound system, indicating enhanced stability upon ligand binding. In the Y260C variant, the pyridoxine-bound complex exhibited multiple shallow minima, indicating higher conformational variability, whereas the TUDCA-bound complex showed a more concentrated and deeper energy basin, suggesting improved structural stability. Overall, the FEL analysis indicates that TUDCA stabilizes the conformational landscape of *AGXT*, particularly in SNP variant systems, by reducing energy dispersion and promoting energetically favorable conformations.

### 2.11. DCCM Results

**Figure 9:**
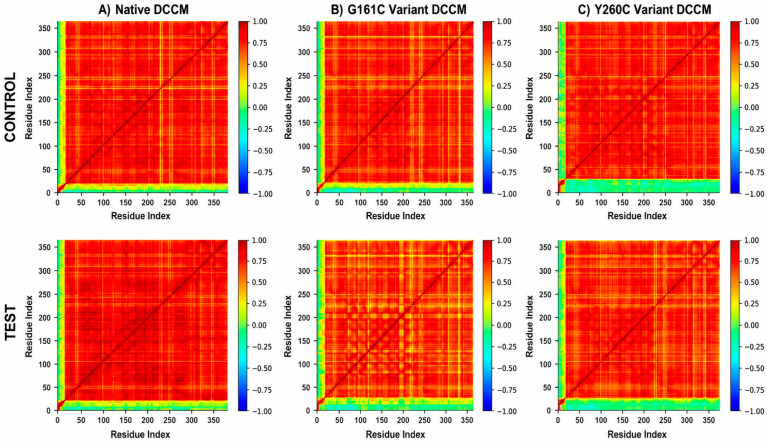
Dynamic cross-correlation matrix (DCCM) plots of native and SNP variant complexes: (**A**) Native, (**B**) G161C variant, and (**C**) Y260C variant under control (top) and test (bottom) conditions, illustrating correlated and anti-correlated residue motions.

The dynamic cross-correlation matrix (DCCM) analysis demonstrated overall coupled motions between native and SNP variant complexes, with predominant positive correlations signifying coordinated residue movements (Figure 9). The native complex exhibited robust and consistent correlations in both the reference drug (control) and test complexes, indicating steady internal dynamics. In the G161C variant, the reference drug complex displayed marginally altered correlation patterns with heightened anti-correlated areas, while the test complex demonstrated more coordinated motions, signifying enhanced dynamic stability upon ligand binding. Likewise, the Y260C variant exhibited significant differences in correlation patterns within the reference drug complex, whereas the test complex demonstrated increased positive correlations and reduced fluctuations. The TUDCA test ligand enhanced residue-level dynamic coordination in both SNP variant complexes, indicating improved stability and coordinated protein motions.

## 3. Discussion

PH1 is an autosomal recessive disease caused by mutations in the *AGXT* gene, leading to oxalate overproduction by the liver-specific peroxisomal enzyme AGT, an enzyme that is critical for normal glyoxylate metabolism. This enzyme is important in glyoxylate metabolism, where it converts glyoxylate to glycine [10]. AGT deficiency increases oxalate production, leading to calcium oxalate crystallization, kidney stones, nephrocalcinosis, and the eventual risk of ESRD. The key to successful management and treatment is early diagnosis and following rigorous management strategies, which may include high volumes of fluid intake and medications and, in cases of severe manifestation, organ transplantation, while ongoing studies are investigating gene therapy and the development of targeted drugs [11].

In this study, an integrated in silico approach was employed to identify pathogenic SNPs and evaluate potential therapeutic candidates. Among the analyzed variants, G161C and Y260C variants were consistently identified as highly deleterious mutations that significantly disrupt *AGXT* structural stability and function, emphasizing the necessity of mutation-specific therapeutic interventions. These variants demonstrated pronounced destabilizing effects on *AGXT* structural integrity, suggesting potential clinical relevance, as such mutations are often associated with protein misfolding, loss of enzymatic function, and increased disease severity in Primary Hyperoxaluria Type 1 [6]. Therefore, G161C and Y260C may represent critical mutation targets for the development of precision-based therapeutic interventions [12].

Virtual screening combined with ADME and toxicity analyses identified Tauroursodeoxycholic acid (TUDCA) as a promising drug candidate with favorable pharmacokinetic and safety profiles. Molecular docking analysis revealed that TUDCA exhibits significantly higher binding affinity compared to the conventional drug pyridoxine across all protein systems, with binding energies of −6.43 kcal/mol (native), −7.58 kcal/mol (G161C), and −5.24 kcal/mol (Y260C). These findings suggest that TUDCA may serve as a potential therapeutic candidate, as its higher binding affinity indicates stronger interaction capability compared to the reference drug. Interaction analysis demonstrated that TUDCA forms stable hydrogen bonds and hydrophobic interactions with key active site residues, such as ARG258, MET259, and TYR54, in the native protein while maintaining strong interactions with SNP variants despite structural alterations. The G161C variant’s highest binding affinity shows that it has the potential to stabilize mutations.

To further validate these interactions, molecular dynamics (MD) simulations over 300 ns demonstrated that TUDCA-bound complexes exhibit enhanced structural stability compared to reference drug systems [13]. These observations indicate that TUDCA may enhance structural stability and mitigate mutation-induced destabilization in *AGXT* variants. Lower RMSD and RMSF values confirmed reduced structural deviations and minimized residue-level fluctuations. Radius of gyration (Rg) and solvent-accessible surface area (SASA) analyses indicated improved compactness and reduced solvent exposure, particularly in SNP variant complexes. Additionally, hydrogen bond analysis showed consistent interaction patterns, with TUDCA maintaining approximately 2–5 hydrogen bonds throughout the simulation, highlighting strong and stable ligand–protein interactions.

Free energy landscape (FEL) analysis further supported these findings by demonstrating that TUDCA-bound systems possess more defined and deeper energy minima, indicating reduced conformational heterogeneity and stabilization of energetically favorable states. The reduced energy dispersion and presence of well-defined minima suggest stabilization of energetically favorable conformational states. This effect was particularly pronounced in SNP variants, where TUDCA reduced energy dispersion and promoted stable conformations. Notably, distinct differences were observed between the G161C and Y260C variants in terms of conformational sampling and stability, suggesting mutation-specific responses to TUDCA binding. Furthermore, Dynamic Cross-Correlation Matrix (DCCM) analysis revealed enhanced positive correlations and improved residue-level coordination in TUDCA-bound complexes, indicating restoration of internal dynamic communication disrupted by the variants [14]. This coordinated residue motion suggests improved internal dynamic stability in TUDCA-bound systems.

This observation is consistent with previous studies demonstrating that Tauroursodeoxycholic acid (TUDCA) functions as a chemical chaperone capable of stabilizing misfolded proteins, reducing endoplasmic reticulum (ER) stress, and preventing protein aggregation [15]. Chemical chaperones such as TUDCA and 4-phenylbutyric acid have been widely reported to restore proteostasis and improve folding efficiency in conformational disorders. In the context of Primary Hyperoxaluria Type 1, *AGXT* mutations are known to induce protein misfolding, aggregation, and mistargeting, ultimately leading to loss of enzymatic activity [16]. Therefore, the enhanced structural stability and improved dynamic behavior observed in TUDCA-bound *AGXT* variants in this study are in alignment with its established role as a protein-stabilizing agent. These findings further support the potential of TUDCA as a mutation-specific therapeutic candidate for correcting structural defects associated with *AGXT* variants.

Importantly, the observed variant-specific differences in stability and interaction patterns suggest the potential for designing TUDCA derivatives tailored to individual SNP variants, thereby supporting a precision medicine approach for PH1.

In conclusion, this study highlights TUDCA as a promising therapeutic candidate capable of stabilizing both native and SNP Variant AGXT proteins. Its superior docking affinity, enhanced dynamic stability, and favorable pharmacological properties suggest a significant advantage over the conventional treatment (pyridoxine). TUDCA appears to function as a chemical chaperone, potentially correcting mutation-induced structural defects and restoring protein stability. While these findings provide strong computational evidence supporting its therapeutic potential, further experimental validation through in vitro and in vivo studies is required. Overall, this study underscores the value of integrating docking, molecular dynamics, and advanced computational analyses in developing precision medicine strategies for PH1.

## 4. Methodology

### 4.1. Dataset and Structure Retrieval

The AGXT protein sequence was obtained in FASTA format from the UniProt database (https://www.uniprot.org/ (accessed on 10 January 2026; UniProt ID: P21549)) [17] and contains 392 amino acids. This sequence served as the basis for subsequent computational analysis. The 3D structure of the AGXT protein was sourced from the Protein Data Bank (https://www.rcsb.org/ (accessed on 10 January 2026; PDB ID: 5F9S)) [17,18] and has a resolution of 1.70 Å, encompassing amino acids 6 to 391. The structure was visualized and preprocessed using PyMOL (version 2.5, Schrödinger LLC, New York, NY, USA), where only the functionally active monomeric A chain was retained to avoid redundancy. All water molecules were removed to eliminate solvent-related artifacts. Subsequent energy minimization was performed using Swiss-PDB Viewer (version 4.1, Swiss Institute of Bioinformatics, Lausanne, Switzerland) by selecting all residues and applying energy refinement to resolve steric clashes and optimize atomic geometry. The minimized structure was then exported for downstream computational studies [17,18]. The SNP variants linked to the AGXT were obtained from publicly accessible online databases such as NCBI (https://www.ncbi.nlm.nih.gov/, accessed on 10 January 2026), UniProt (https://www.uniprot.org/, accessed on 10 January 2026), and HGMD (http://www.hgmd.cf.ac.uk/, accessed on 10 January 2026). The literature study of these variants was performed utilizing PubMed (https://pubmed.ncbi.nlm.nih.gov/, accessed on 10 January 2026), ScienceDirect (https://www.sciencedirect.com/, accessed on 10 January 2026), and ResearchGate (https://www.researchgate.net/, accessed on 10 January 2026) [17,18].

### 4.2. Evolutionary Analysis

Evolutionary analysis was carried out to identify the conserved amino acid regions in the *AGXT* using the ConSurf web server (https://consurf.tau.ac.il/, accessed on 15 January 2026) [19]. This helps in identifying the critical regions that are crucial for the protein’s functional activity. The FASTA sequence served as the input for the AGXT protein, and the output from ConSurf provided conservation scores for every residue, scoring them from 0 (indicating minimally conserved, inferring high evolutionary variability and tolerance to substitutions) to 9 (indicating highly conserved, strong evolutionary constraint due to structural and functional importance) [20]. Conserved residues with a score of 9, representing the most evolutionarily constrained sites, were selected for further analysis.

### 4.3. Biochemical Analysis Using Align-GVGD

Biophysical properties of various classes of amino acids, including their hydrophobic, hydrophilic, aromatic, heterocyclic, acidic, or basic characteristics at neutral pH, are examined to determine the likelihood of amino acid substitution [17]. The Align-GVGD tool (http://agvgd.hci.utah.edu/, accessed on 15 January 2026) is utilized to predict the functional impact of substitutions, especially of evolutionarily distant residues. The input for this analysis includes the FASTA sequence and a curated list of SNP variants obtained from evolutionary conservation analysis. The output from the Align-GVGD tool provided Grantham Variation (GV) scores, which quantify how much a position can tolerate substitutions, and Grantham Deviation scores (GD), which evaluate how much a specific amino acid substitution differs from the original [21]. Variants with the highest GD scores, categorized under the C65 class, were selected for further analysis due to their strong predicted deleterious impact on *AGXT* function. In contrast, variants in the C0 category were considered least likely to affect protein function, while intermediate classes represented a gradual increase in the potential for functional disruption [22].

### 4.4. Analysis of Structural Stability

Stability analysis is critical for determining whether SNP variants are stabilizing or destabilizing to their structure. This analysis uses several computational tools including INPS-MD, CUPSAT, and iStable, to analyze the impact of deleterious variants on the AGXT protein. INPS-MD is a sequence-based predictor that estimates stability changes (ΔΔG) associated with non-synonymous mutations [23]. It utilizes a support vector machine (SVM) regression model trained on a dataset containing experimentally validated mutations. The tool incorporates sequence-derived features such as amino acid substitution scores, hydrophobicity, mutability, molecular weights, and evolutionary conservation scores derived from hidden Markov models. It requires the protein sequence in FASTA format and a list of SNP variants as input, and the tool outputs predicted free energy change (ΔΔG) in kcal/mol. Positive ΔΔG values indicate increased stability, while negative ΔΔG values indicate destabilization [24].

CUPSAT (http://cupsat.tu-bs.de, accessed on 18 January 2026) (Cologne University Protein Stability Analysis Tool) http://cupsat.tu-bs.de (accessed on 18 January 2026) is a structure-based tool for predicting the effect of single amino acid mutations on protein stability. It incorporates torsion angle potentials, solvent accessibility, and secondary structure preferences to derive the free energy difference (ΔΔG) between native and SNP variant proteins. It requires a protein’s 3D structure and variant details as input. The output presents the predicted ΔΔG in kcal/mol, with positive values for stabilizing mutations and negative for destabilization [25].

iStable (http://predictor.nchu.edu.tw/istable, accessed on 18 January 2026) (integrated Stability prediction) http://predictor.nchu.edu.tw/istable (accessed on 18 January 2026) is a meta-predictor that enhances accuracy by combining several stability prediction tools, like I-Mutant and MUpro. It uses sequence and structure-based features to predict the effect of single amino acid substitutions on protein stability via a two-layer prediction model [21]. iStable can use either a protein sequence in FASTA format or a 3D structure in PDB format and variant information (wild-type amino acid, position, and mutant amino acid) as input. The output gives the predicted ΔΔG value in kcal/mol, where a positive value indicates increased stability while a negative value indicates destabilization. The results feature a reliability score and individual predictors’ contributions, presented on a web interface or downloadable as a text file [26].

### 4.5. SNP Variant Structure Preparation by Mutagenesis

Site-directed mutagenesis was performed using PyMOL (version 2.5, Schrödinger LLC, New York, NY, USA) to evaluate the effect of mutations on the AGXT protein. The refined and energy-minimized wild-type AGXT protein structure was imported into PyMOL, and the target amino acids were substituted with the desired mutated residues [22,27]. The variant structures were then energy-minimized to adjust atomic positions and stabilize the altered conformation. The resulting SNP variant structures were subsequently saved in PDB format, allowing for further computational analysis.

### 4.6. Ligand Retrieval and Preparation

To identify the potential drug candidates for repurposing in PH1, a comprehensive search was carried out utilizing literature databases and the DrugBank database (https://go.drugbank.com/, accessed on 20 January 2026), focusing exclusively on FDA-approved compounds [23]. The selection criteria were based on pharmacological relevance to PH1-associated symptoms, such as hyperoxaluria, kidney stone formation, and hepatic metabolic dysfunction. This approach yielded a total of 111 candidate compounds. Structural and pharmacological data for these compounds were retrieved from the PubChem database (https://pubchem.ncbi.nlm.nih.gov/, accessed on 20 January 2026), with canonical SMILES and associated bioactivity information collected to ensure standardized representation. The 3D conformers of all selected ligands were downloaded in SDF format to facilitate uniform input for virtual screening, molecular docking, and molecular dynamics simulations. The resulting curated and standardized dataset ensured consistency and reliability in subsequent computational analyses.

### 4.7. Analysis of Protein Binding Sites

Potential ligand-binding pockets in the AGXT protein were identified using the CASTp server (http://sts.bioe.uic.edu/castp/, accessed on 20 January 2026), which analyzes protein surface topography to detect concave regions suitable for ligand interaction. Upon submitting the AGXT protein structure (PDB ID: 5F9S), CASTp provided data on surface area, volume, and constituent amino acid residues of predicted binding pockets [27]. These insights guided the selection of target sites for subsequent molecular docking analyses.

### 4.8. Virtual Screening

Virtual screening of the 111 selected FDA-approved compounds against the AGXT protein was performed using PyRx, an integrated virtual screening tool that combines AutoDock Vina and Open Babel for efficient molecular docking workflows. The energy-minimized AGXT protein structure was imported and converted into PDBQT format, while ligand structures were batch-processed and prepared similarly using Open Babel within PyRx [17]. Docking was carried out using AutoDock Vina, with a grid box defined around the predicted active site to restrict docking to the biologically relevant region. Binding affinities were evaluated based on calculated binding energy scores, and top-ranked compounds with the lowest binding energies were shortlisted for further analysis [17,28].

### 4.9. ADME Analysis

ADME properties of the selected compounds were evaluated using the SwissADME (http://www.swissadme.ch/, accessed on 22 January 2026). Canonical SMILES were submitted to obtain pharmacokinetic descriptors, including physicochemical properties, lipophilicity (Log P), solubility, gastrointestinal absorption, blood–brain barrier permeability, and cytochrome P450 interactions. Drug-likeness was assessed based on Lipinski’s Rule of Five, and compounds without violations were considered suitable for further analysis [28]. The canonical SMILES of shortlisted compounds were then retrieved from the PubChem database for subsequent toxicity evaluation.

### 4.10. Toxicity Analysis

Mcule platform (https://mcule.com, accessed on 25 January 2026) is an advanced online platform for molecular modeling, drug discovery, and toxicity prediction. The canonical SMILES structure of each compound was used as an input for this tool. Based on the molecular structure, Mcule produced a detailed toxicity profile, providing predictions regarding potential carcinogenicity, hepatotoxicity, mutagenicity, and reproductive toxicity [28,29]. The toxicity scores obtained from Mcule were retained for further analysis and subsequently compared with the results from Protox-3 to ensure consistency in toxicity classification.

Toxicity analysis was performed using ProTox-3 (https://tox-new.charite.de/protox_III/, accessed on 25 January 2026), which employs machine learning and molecular fingerprints for accurate prediction of toxicological endpoints, including acute toxicity, carcinogenicity, hepatotoxicity, and immunotoxicity. Canonical SMILES of the compounds were used as input for analysis. Toxicity was expressed as LD50 (mg/kg), representing the median lethal dose [30]. Compounds were classified according to the Globally Harmonized System (GHS) into Classes I–VI, ranging from highly toxic (Class I) to non-toxic (Class VI). These classifications enabled systematic evaluation of compound safety, with higher classes indicating lower toxicity [31].

### 4.11. Molecular Docking

Molecular docking studies were conducted to investigate the interaction between the AGXT protein and selected ligands using AutoDock 4 (The Scripps Research Institute, La Jolla, CA, USA) [32,33,34]. The protein structure was prepared by uploading it into AutoDock, adding polar hydrogens, assigning Kollman charges to the protein, and saving it as a PDBQT file [28,30]. Ligand preparation involved importing the molecules, adding polar hydrogens, assigning Gasteiger charges, building a torsion tree to define rotatable bonds for flexibility, and saving the ligands in PDBQT format. The active site of the AGXT protein was identified by manually selecting residues from the protein’s active site, setting up a grid box to cover the binding site, and generating grid parameter files using AutoGrid4 [35]. Docking was performed with the Genetic Algorithm (GA) search method, specifically using the Lamarckian Genetic Algorithm (LGA), and results were analyzed by ranking docking conformations based on binding energy [36]. The most favorable poses were identified by evaluating binding energies, visualized using Discovery Studio Visualizer, and further analyzed for molecular interactions such as hydrogen bonding, hydrophobic, and electrostatic interactions [37]. A 2D interaction diagram was generated to pinpoint key residues involved in binding, and the ligand–protein interactions were studied through molecular surface representations and distance measurements, which provided additional structural insights into the docking process and supported the observed binding affinities [38].

### 4.12. Molecular Dynamics Simulation

Molecular Dynamics (MD) simulations were performed using GROMACS (version 2025.1, University of Groningen, Groningen, The Netherlands) to evaluate the structural stability and dynamic behavior of protein–ligand complexes, including both wild-type *AGXT* and SNP variants (G161C and Y260C) in complex with the TUDCA compound, Tauroursodeoxycholic acid, and pyridoxine used as a reference drug (therapeutic standard), for a 300 ns simulation [17]. The GROMOS96 54a7 force field was applied, and ligand topology files were generated using the Automated Topology Builder (ATB) v3.0. The protein–ligand complex was solvated within a cubic simulation box using a Simple Point Charge (SPC) water model, with the box edge maintained at least 1.0 nm away from any atom of the complex. The system was neutralized by adding the appropriate amount of counterions (Na^+^ or Cl^−^), followed by energy minimization using the steepest descent method to remove any unfavorable contacts or steric clashes. Two equilibration stages were then performed: NVT equilibration for 100 ps to stabilize the system’s temperature at 300 K using a V-rescale thermostat, and NPT equilibration for 100 ps to stabilize pressure at 1 bar using the Parrinello–Rahman barostat. After equilibration, production MD simulations were conducted for 300 ns for each complex under periodic boundary conditions. Trajectory data were stored in .xtc files at regular intervals for further analysis. Several structural and thermodynamic parameters were calculated to assess the conformational stability and dynamics of the complexes, including Root Mean Square Deviation (RMSD) using gmx rms, Root Mean Square Fluctuation (RMSF) using gmx rmsf, Radius of Gyration (Rg) using gmx gyrate, intermolecular hydrogen bonds using gmx hbond, and Solvent Accessible Surface Area (SASA) using gmx sasa. The simulations were repeated three times to ensure consistency, and for final analysis, the average of each parameter was considered. The resulting data were then plotted to assess the dynamics and stability of the protein–ligand complexes over the course of the simulation.

### 4.13. Free Energy Landscape (FEL) Analysis

The free energy landscape (FEL) analysis was performed by mapping the molecular dynamics trajectories onto the first two principal components (PC1 and PC2) derived from principal component analysis [28,30,39]. To facilitate direct comparison, the FELs were constructed with uniform binning and plotting parameters across all systems. The resultant landscapes were employed to visualize the distribution of conformational states gathered during simulations and to identify low-energy regions corresponding to the stable conformation of protein–ligand interactions.

### 4.14. Dynamic Cross-Correlation Matrix (DCCM) Analysis

DCCM analysis was performed on the equilibrated segments of the MD trajectories to analyze ligand-induced alterations in long-range residue-residue communication and intracellular protein dynamics [28,30,39,40]. Correlations were determined with Cα atom fluctuations by the formula;

Cij = ❬Δri · Δrj❭/[❬Δri^2^❭ ❬Δrj^2^❭]½

where Δri and Δrj denote the positional variations in residues i and j in respect to their mean positions. The correlation coefficient varies from −1 to +1, suggesting a spectrum from complete anti-correlation to complete correlation. Reference drug, test, and differential DCCM maps were generated with equal correlation thresholds.

## 5. Conclusions

This study demonstrates that Tauroursodeoxycholic acid (TUDCA) is a promising therapeutic candidate for Primary Hyperoxaluria Type 1 (PH1), particularly in targeting deleterious *AGXT* variants such as G161C and Y260C. TUDCA exhibited superior binding affinity compared to pyridoxine and maintained stable interactions with both native and SNP variant proteins. Molecular dynamics simulations further confirmed its ability to enhance structural stability by reducing fluctuations, improving compactness, and sustaining consistent hydrogen bonding. Additionally, FEL and DCCM analyses indicated improved conformational stability and coordinated residue dynamics in TUDCA-bound systems. Overall, these findings suggest that TUDCA may help stabilize variant-induced structural defects in *AGXT*, highlighting its potential as a variant-specific therapeutic agent. However, these results are based on computational analyses, and further experimental validation through in vitro and in vivo studies is essential to confirm its therapeutic efficacy and clinical applicability in PH1 treatment.

## Figures and Tables

**Table 1:** Retrieval of AGXT protein sequence and corresponding structural data from.

Protein	Uniprot ID	PDB ID	Resolution	Structure
*AGXT*	P21549	5F9S	1.70 Å	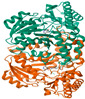

**Table 2:** Compilation of *AGXT* SNP variants obtained from various genomic databases.

Databases	No. of Variants	Total SNPs
Uniprot	41	261
HGMD	63	
ClinVar	133	
PubMed	11	
Research Gate	13	

**Table 3:** Active site residues of *AGXT* identified using CASTp.

Structure	AMINO ACID RESIDUES	PocID	Area (SA) Å^2^	Volume (SA) Å^3^
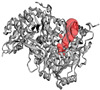	48SER, 49MET, 50SER, 51LYS, 54TYR, 250PHE, 257PRO, 258ARG, 259MET, 260TYR, 261HIS, 262HIS, 263THR, 264ILE	1	628.095	600.179

**Table 4:** CUPSAT stability prediction of *AGXT* SNP variants.

SNP VARIANTS	CUPSAT
S81W	Destabilizing
G82E	Stabilizing
G82L	Stabilizing
W108R	Stabilizing
W108C	Destabilizing
R118P	Stabilizing
S158L	Stabilizing
G161C	Destabilizing
Y260C	Destabilizing
L276E	Stabilizing
L276Q	Stabilizing

**Table 5:** iStable-based stability analysis of selected *AGXT* SNP variants.

Predictor	i-Mutant2.0 SEQ	DDG	MUpro	Conf. Score	iStable	Conf. Score
S81W	null	null	Increase	0.39261706	Increase	0.735838
W108C	null	null	Increase	0.049000388	Increase	0.700978
G161C	null	null	Decrease	−0.033219596	Decrease	0.709536
Y260C	null	null	Decrease	−1	Decrease	0.678621

**Table 6:** Toxicity class prediction of selected compounds using ProTox-3 based on LD_50_ values.

Compound ID	Predicted LD50 (mg/kg)	Predicted Toxicity Class
214348	1000	Class 4
9848818	5000	Class 5
10133	2000	Class 4
2315	10,000	Class 6
2732	5000	Class 5
158781	298	Class 3
134018	8000	Class 6
4170	5000	Class 5
60150535	800	Class 4
4578	800	Class 4
5546	285	Class 3
3961	300	Class 3
60846	2000	Class 4
156391	248	Class 3
2471	4624	Class 5
3440	2000	Class 4
5329	2300	Class 5

LD_50_: Median lethal dose; mg/kg: milligrams per kilogram.

**Table 7:** Molecular docking results of test and reference compounds against AGXT protein variants.

Protein Type	Reference Drug (Pyridoxine) Binding Affinity (kcal/mol)	Test (Tauroursodeoxycholic Acid) Binding Affinity (kcal/mol)
Native Protein	−2.96	−6.43
SNP Variant G161C	−5.22	−7.58
SNP Variant Y260C	−4.97	−5.24

## Data Availability

All data generated or analyzed during this study are included in this published article (and its supplementary information files).

## Supplementary Materials

The following supporting information can be downloaded at: https://www.mdpi.com/article/10.3390/ijms27104590/s1.
