# Supplementary material for: Targeting the Highly Deleterious G161C and Y260C SNP Variants of the AGXT Protein Involved in Glyoxylate Metabolism Using Tauroursodeoxycholic Acid: A Computational Study
Source: Int J Mol Sci. 2026 May 20;27(10):4590. doi: 10.3390/ijms27104590 (PMC13206818; doi:10.3390/ijms27104590)
Supplement: Supplementary file 1 [file ijms-27-04590-s001.zip › ijms-4253779-supplementary.pdf]

Targeting the Highly Deleterious G161C and Y260C SNP Variants of the AGXT Protein Involved in Glyoxylate Metabolism Using Tauroursodeoxycholic Acid: A Computational Study

Shruthika G[1#], Vasundra V[1#], Sidharth Kumar N[1], Madhana Priya N[2], and Magesh R[1*]**

[1] Department of Biotechnology, Faculty of Biomedical Sciences & Technology, Sri Ramachandra Institute of Higher Education and Research (DU), Chennai.

[2] Center for Transdisciplinary Research, Department of Pharmacology, Saveetha Dental College and Hospitals, Saveetha Institute of Medical and Technical Sciences, Chennai 600077, Tamil Nadu, India

# Authors contributed equally to this work.

* Corresponding Author (Email: magesh.r@sriramachandra.edu.in)

** Co-Corresponding Author (Email: madhanapriyan.sdc@saveetha.com)

* Correspondence: Dr. Magesh Ramasamy, Department of Biotechnology, Sri Ramachandra Institute of Higher Education and Research (DU), Porur 600116, Chennai, Tamil Nadu, India. Email: magesh.r@sriramachandra.edu.in

**Co-correspondence: Dr. Madhana Priya N, Center for Transdisciplinary Research, Department of Pharmacology, Saveetha Dental College and Hospitals, Saveetha Institute of Medical and Technical Sciences, Chennai 600077, Tamil Nadu, India. Email: madhanapriyan.sdc@saveetha.com

Table S1. Biophysical classification of AGXT variants using Align-GVGD

Substitution	GV	GD	Prediction
L25R	0	101.88	Class C65
G27W	0	183.79	Class C65
G27E	0	97.85	Class C65
P28S	0	73.35	Class C65
G47R	0	125.13	Class C65
G47E	0	97.85	Class C65
T70N	0	64.77	Class C55
N72D	0	23.01	Class C15
N72H	0	68.35	Class C65
N72K	0	93.88	Class C65
N72I	0	148.91	Class C65
T75K	0	77.74	Class C65
S81W	0	176.58	Class C65
S81L	0	144.08	Class C65
G82E	0	97.85	Class C65
G82R	0	125.13	Class C65
G82L	0	137.69	Class C65
G82A	0	60	Class C55
E95K	0	56.87	Class C55
E95D	0	44.6	Class C35
G97R	0	125.13	Class C65
D98H	0	81.24	Class C65
W108R	0	101.29	Class C65
W108C	0	214.36	Class C65
R111Q	0	42.81	Class C35
R118S	0	109.21	Class C65
R118C	0	179.53	Class C65
R118P	0	102.71	Class C65
R118H	0	28.82	Class C25
G156R	0	125.13	Class C65
G156A	0	60	Class C55
E157Q	0	29.27	Class C25
S158L	0	144.08	Class C65
G161C	0	158.23	Class C65

G161R	0	125.13	Class C65
G161S	0	55.27	Class C55
G161D	0	93.77	Class C65
Q164R	0	42.81	Class C35
Q164L	0	112.44	Class C65
D183N	0	23.01	Class C15
A186V	0	64.43	Class C55
S187Y	0	143.11	Class C65
S187F	0	154.81	Class C65
D201N	0	23.01	Class C15
D201V	0	152.01	Class C65
D201E	0	44.6	Class C35
Q208E	0	29.27	Class C25
K209N	0	93.88	Class C65

Table S2. INPS-MD stability prediction of AGXT SNP variants

SNP VARIANTS	PREDICTION
L25R	Neutral
G27W	Stabilizing
G27E	Neutral
P28S	Neutral
G47R	Neutral
G47E	Weakly destabilizing
N72H	Neutral
N72K	Neutral
N72I	Neutral
T75K	Weakly destabilizing
S81W	Destabilizing

S81L	Weakly destabilizing
G82E	Destabilizing
G82R	Weakly destabilizing
G82L	Destabilizing
G97R	Neutral
D98H	Neutral
W108R	Destabilizing
W108C	Destabilizing
R118S	Weakly destabilizing
R118C	Weakly destabilizing
R118P	Destabilizing
G156R	Weakly destabilizing
S158L	Destabilizing
G161C	Destabilizing
G161R	Neutral
G161D	Neutral
Q164L	Stabilizing
S187Y	Neutral
S187F	Neutral
D201V	Neutral
K209N	Neutral
G216R	Neutral
R233C	Weakly destabilizing
R233L	Neutral
D243H	Neutral
R258S	Weakly destabilizing
Y260C	Destabilizing
H261D	Weakly destabilizing
L276E	Destabilizing

L276Q	Destabilizing
H291P	Neutral
R317W	Weakly destabilizing
G350D	Weakly destabilizing
R360P	Neutral
R360W	Neutral
G365C	Neutral
G365D	Weakly destabilizing

Table S3. Virtual Screening of top 50 drug candidates using PyRx

Ligand	Binding Affinity	rmsd/ub	rmsd/lb
5f9s_em-1_24812758_uff_E=551.27	-7.1	0	0
5f9s_em-1_443894_uff_E=774.68_uff_E=774.03	-7	0	0
5f9s_em-1_9848818_uff_E=1004.97	-6.9	0	0
5f9s_em-1_214348_uff_E=648.32	-6.8	0	0
5f9s_em-1_443872_uff_E=2172.42	-6.8	0	0
5f9s_em-1_135398658_uff_E=289.04	-6.8	0	0
5f9s_em-1_10133_uff_E=543.37	-6.7	0	0
5f9s_em-1_2315_uff_E=1087.57	-6.7	0	0
5f9s_em-1_11949646_uff_E=511.71	-6.6	0	0
5f9s_em-1_5833_uff_E=705.02	-6.6	0	0
5f9s_em-1_126941_uff_E=306.25	-6.5	0	0
5f9s_em-1_65999_uff_E=1129.40	-6.5	0	0
5f9s_em-1_9887712_uff_E=352.74	-6.3	0	0
5f9s_em-1_5281104_uff_E=540.81	-6.2	0	0
5f9s_em-1_3702_uff_E=893.60	-6.1	0	0

5f9s_em-1_2732_uff_E=707.66	-6	0	0
5f9s_em-1_444493_uff_E=2804.46	-6	0	0
5f9s_em-1_5280453_uff_E=652.31	-6	0	0
5f9s_em-1_5281107_uff_E=613.97	-6	0	0
5f9s_em-1_158781_uff_E=1119.99	-5.9	0	0
5f9s_em-1_2993_uff_E=423.64	-5.9	0	0
5f9s_em-1_5282242_uff_E=1396.98	-5.9	0	0
5f9s_em-1_54671203_uff_E=690.46	-5.9	0	0
5f9s_em-1_3749_uff_E=1130.97	-5.9	0	0
5f9s_em-1_134018_uff_E=445.73	-5.8	0	0
5f9s_em-1_4170_uff_E=805.63	-5.8	0	0
5f9s_em-1_5362129_uff_E=546.94	-5.8	0	0
5f9s_em-1_60150535_uff_E=511.24	-5.8	0	0
5f9s_em-1_2764_uff_E=1585.31	-5.7	0	0
5f9s_em-1_42613186_uff_E=650.57	-5.7	0	0
5f9s_em-1_4578_uff_E=393.43	-5.6	0	0
5f9s_em-1_5546_uff_E=311.95	-5.6	0	0
5f9s_em-1_3961_uff_E=1052.15	-5.6	0	0
5f9s_em-1_6321411_uff_E=1444.76	-5.6	0	0
5f9s_em-1_14985_uff_E=288.57	-5.5	0	0
5f9s_em-1_5388962_uff_E=313.91	-5.5	0	0
5f9s_em-1_60846_uff_E=669.60	-5.5	0	0
5f9s_em-1_129211_uff_E=694.14	-5.4	0	0
5f9s_em-1_156391_uff_E=140.28	-5.4	0	0
5f9s_em-1_41781_uff_E=650.52	-5.4	0	0
5f9s_em-1_5284603_uff_E=563.95	-5.4	0	0

5f9s_em-1_71511839_uff_E=1206.45	-5.3	0	0
5f9s_em-1_2471_uff_E=680.22	-5.2	0	0
5f9s_em-1_4740_uff_E=394.40	-5.2	0	0
5f9s_em-1_5988_uff_E=487.38	-5.2	0	0
5f9s_em-1_6167_uff_E=1040.23	-5.2	0	0
5f9s_em-1_156419_uff_E=252.81	-5	0	0
5f9s_em-1_3440_uff_E=761.63	-5	0	0
5f9s_em-1_3639_uff_E=948.06	-5	0	0
5f9s_em-1_5329_uff_E=724.00	-5	0	0

Table S4. Pharmacokinetic and drug-likeness properties of selected compounds predicted by SwissADME

Compound ID	Energy Score (uff_E)	ADME Violation Value
24812758	551.27	0
9848818	1004.97	0
214348	648.32	0
443872	2172.42	0
10133	543.37	0
2315	1087.57	0
11949646	511.71	0
5833	705.02	0
9887712	352.74	0
3702	893.60	0
2732	707.66	0
158781	1119.99	0
5282242	1396.98	0

134018	445.73	0
4170	805.63	0
5362129	546.94	0
60150535	511.24	0
2764	1585.31	0
4578	393.43	0
5546	311.95	0
3961	1052.15	0
5388962	313.91	0
60846	669.60	0
129211	694.14	0
156391	140.28	0
41781	650.52	0
5284603	563.95	0
2471	680.22	0
4740	394.40	0
6167	1040.23	0
3440	761.63	0
3639	948.06	0
5329	724.00	0

Table S5. Prediction of toxicity profiles of selected compounds using MCULE for drug safety evaluation

Compound ID	Mcule Toxicity
24812758	TOXIC
9848818	NON-TOXIC

214348	NON-TOXIC
443872	TOXIC
10133	NON-TOXIC
2315	NON-TOXIC
11949646	TOXIC
5833	TOXIC
9887712	TOXIC
3702	TOXIC
2732	NON-TOXIC
158781	NON-TOXIC
5282242	TOXIC
134018	NON-TOXIC
4170	NON-TOXIC
5362129	TOXIC
60150535	NON-TOXIC
2764	TOXIC
4578	NON-TOXIC
5546	NON-TOXIC
3961	NON-TOXIC
5388962	TOXIC
60846	NON-TOXIC
129211	TOXIC
156391	NON-TOXIC
41781	TOXIC
5284603	TOXIC
2471	NON-TOXIC

4740	TOXIC
6167	TOXIC
3440	NON-TOXIC
3639	TOXIC
5329	NON-TOXIC
